# Pro–poor pathway towards universal health coverage: lessons from Ethiopia

**DOI:** 10.7189/jogh.06.010305

**Published:** 2016-06

**Authors:** Kesetebirhan Admasu, Taye Balcha, Tedros Adhanom Ghebreyesus

**Affiliations:** 1 Ministry of Health, Addis Ababa, Ethiopia; 2 Ministry of Foreign Affairs, Addis Ababa, Ethiopia

Protection from care–related catastrophic expenditures through equitable access to affordable health services is the hallmark of a pro–poor health policy [1]. Over the past two decades, the Government of Ethiopia has implemented policies with a clear intent of reducing poverty and improving the daily lives of its citizens, especially the poor [2]. Guided by these cross cutting pro–poor government policies and spurred by the United Nations Millennium Development Goals (MDGs), the health sector has implemented multi–pronged initiatives towards ensuring every citizen an access to affordable health services without catastrophic expenditures (**Table 1**). The health sector initiatives have been guided by evolution of the innovative health programs nationally introduced as well as the needs of the community in each village across the country [3,4]. Primary focus on the poor and ownership of new health initiatives by the community have been the linchpin for investment at scale.

**Table 1:** Major pro–poor policies and initiatives in Ethiopia

Pro–poor initiatives	Year implemented	Objective	Key outcomes
Introduction of Health Extension Program	2003	To achieve universal primary health care coverage which mainly benefits low–income households	More than 38 000 health extension workers have been deployed in 16 500 villages of the country; and universal primary health care coverage has been achieved.
Establishment of Pharmaceuticals Fund and Supply Agency	2006	To ensure accessibility and affordability of essential medicines and laboratory investigations	Medicines and laboratory investigations for key health conditions have been provided free of charge; out–of–pocket expenditures have reduced; health services utilization has improved; and health MDG targets have been met.
Health Development Army Program with community soolidarity fuding	2012	To disseminate health information and facilitate uptake of critical health services and finance priority challenges identified by the community	Procured more than 200 ambulance vehicles for medical referral; constructed health posts and maternity waiting homes at rural health centers; and Health Development Armies have actively involved in health facility governance to improve the quality of health services.
Scaling up Community–based Health Insurance scheme	2015	To provide quality health care without financial hardship to the poor in informal sector	By the end of 2016, 50% of citizens in informal sector are expected to be covered.
Implementation of Social Health Insurance scheme	2016	To deliver quality health care and ensure financial protection to citizens employed in formal sector and achieve universal health coverage	All employees of formal sector are expected to be covered by the end of 2016.

All–inclusive, community–led primary health care is the bedrock of the health services in Ethiopia. Notwithstanding its low–income status, the country has gradually but radically expanded access to a spectrum of health services and essential medicines. More than 38 000 all–female Health Extension Workers (HEWs) have been deployed to more than 16 500 community health posts across the country to lead a novel primary health care– Health Extension Program (HEP). Launched in 2003, the HEP has brought simple, cost–effective and locally–desired health interventions [4] close to where the country’s majority, rural citizens live. The service package includes maternal and child health (family planning, antenatal services, immunization, nutrition services, treatment of infectious childhood conditions), prevention, and sometimes treatment of communicable diseases (tuberculosis, HIV and malaria) and environmental sanitation. All health services provided by HEWs at health post level are free of charge. The government carries the brunt of the financial costs for these services but it is commonly supplemented by innovative community financing. For example, while the government has assigned trained and salaried HEWs as civil servants in each village and provided ambulance vehicles at district level, the community has constructed health posts and maternity waiting homes at health centers, and has procured additional ambulance vehicles for medical referral through Health Development Army [3]. The new government–community partnership has improved service uptake and health outcomes. A study in Northern Ethiopia attributed a substantial reduction in pregnancy–related mortalities to a wide availability of ambulance services for obstetric referral [5].

Eyeing Universal Health Coverage (UHC), Ethiopia has intensified its implementation of pro–poor initiatives. It is currently rolling out the second generation HEP to meet the growing needs of the community. The second generation program adds more interventions targeted on emerging infectious diseases, common non–communicable diseases and, mental health. It also includes deepening the partnership with the community even beyond primary care level. The government has put a mechanism to stimulate innovations improving access to and utilization of health services by the poor [6].

Whereas the HEP remains country’s priority, considerable efforts have been made to improve the higher levels of health care. For example the number of health centers and hospitals in the country has sharply increased by 350% and 150%, respectively between 2004 and 2015. Correspondingly, an enormous development has been made in human resources for health through increasing the number of medical universities. Tuition fees and lodging expenses have been covered by the government in all public medical universities and colleges; and a pay–by–service strategy has been implemented whereby health professionals eventually return the cost by rendering health services at public health facilities. As a consequence, the number of medical doctors graduated annually in the country greatly increased, from about 150 in 2004 to 3000 in 2016. Similarly, unprecedented increase has been seen in the number of other critical cadres including mid–wives and specialized nurses. To achieve the right balance between public health and clinical interventions, the country trained innovative cadres like public health officers and integrated emergency surgical officers. Integrated emergency surgical officers perform major surgeries for emergency obstetric and surgical conditions, close to where the rural poor live [7].

In 2006, Ethiopia established Pharmaceutical Fund and Supply Agency (PFSA) with the primary mission of expanding access to medicines, vaccines and laboratory services. Following its launch, PFSA has steered a comprehensive health supply chain management in the country. The agency has used pooled procurement strategy as a vantage to gain economies of scale. It has procured refrigerated trucks, constructed 17 cold room–installed hubs, at least one within 180 km radius of each health facility across the country to maximize the efficiency gains in pharmaceuticals distribution. These gains in efficiency have led to a huge decline in retail prices of medicines with consequential drop in the out–of–pocket expenditure [8], benefiting particularly the poor. Further, the total value of PFSA’s products has sharply grown from US$ 100 million in 2008 to US$ 1.3 billion in 2015; it is projected to be more than US$ 2 billion by 2017.

A complete set of care for priority maternal and child health interventions (family planning, abortion care, labour and delivery, immunization and nutrition services), infectious diseases (tuberculosis, malaria and HIV) and diseases of poverty (onchocerciasis, podoconiosis and trachoma) are provided free of charge at all public service delivery points. Community needs, the ability to pay and potential population level impacts were factored in defining program services and medicines. The strategic removal of financial barrier to care for maternal and child conditions and major infectious diseases has significantly contributed to Ethiopia’s recent achievements including meeting all health MDG targets.

Improved access to affordable health care embraces the budding non–communicable diseases including diabetes mellitus and cardio–vascular diseases. For instance, mark–up has been removed from insulin and medicines used for common malignant conditions. To address the growing demand and expectations of the public and the limited outlets for medicines and supplies for non–prioritised interventions, Ethiopia is currently rolling out community pharmacies in cities and big towns across the country. Similar to health facility–embedded pharmacies, community pharmacies provide medicines at substantially reduced prices.

Ethiopia has established a stringent regulatory system to guard the poor against low standard and counterfeit medicines. Evidence shows that the comprehensive supply management and robust regulation has effectively blocked entry of counterfeit medicines into the market and significantly reduced stock–outs and wastage of medicines [9]. Furthermore, it has improved the affordability of medicines with consequential upsurge in health care utilization. In 2013, the availability of essential medicines at public health facilities in Ethiopia was 76%. The average price of generic medicines significantly reduced between 2004 and 2013, positively impacting the poor. During the same period, a considerable drop in price of medicines at private for–profit outlets was reported [10], implying the strategy’s proxy impact on price regulation in the country’s health care market, further contributing to the mitigation of catastrophic expenditures on the poor.

**Figure:**
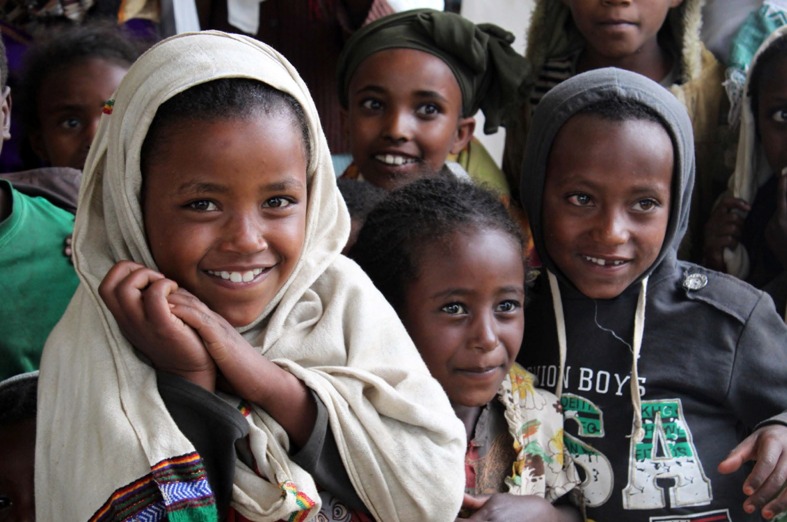
Photo: Children at a vaccinations clinic near Sululta, Ethiopia. Yasmin Abubeker/DFID [CC BY-SA 2.0 (http://creativecommons.org/licenses/by-sa/2.0)], via Wikimedia Commons

Although access to health care has been expanded, significant geographic disparities persist in regards to health care utilization and health outcomes. The inequity in health care is largely attributed to the lifestyles of communities. Pastoralist communities in Ethiopia are generally lagging behind in major health indicators. Further, differences in service uptake exist within communities, denting Ethiopia’s pro–poor, pro–equity route towards UHC.

The health sector is currently providing differentiated support to communities and regions left behind. More broadly, to ensure the universality of health coverage and prevent financial impoverishment, it is scaling up the successful Community–Based Health Insurance pilot for citizens in informal sector, where the poor predominate. Also, the country is introducing Social Health Insurance targeted at employees of formal sector for full coverage. Both schemes offer a package of clinical services without cost ceiling upon services provided at any domestic health facility. The two schemes are expected to cover 80% of the population within the next 5 years and will be consolidated as a single payer system within the next 10 years.

In conclusion, Ethiopia advances locally–tailored, multi–faceted pro–poor approaches to ensure UHC building on its successful transformation of the health sector in the last two decades and the achievement of key health MDG targets. Broader plan for inclusive economic development, effective implementation of primary health care, expansion of access to medicines and introduction of health insurance is the pathway towards UHC for all citizens. Concurrently, quality improvement initiatives and pushing non–communicable diseases to the forefront of the agenda are under way. More importantly, levelled partnership of the government with the community across the spectrum of health care ensures sustainability and community ownership of the system. We believe that these approaches to health care could propel Ethiopia to expedite its efforts to achieve the sustainable development goals including UHC within a short time.

## References

[1] DFID Health Systems Resource Centre. Health financing: designing and implementing pro–poor policies. London: DFID HSRC, 2001. Available: http://www.heart–resources.org/wp–content/uploads/2012/10/Health–financing.pdf. Accessed: 8 March 2016.

[2] Ministry of Finance and Economic Development of Ethiopia. Building on Progress: A Plan for Accelerated and Sustained Development to End Poverty (PASDEP). Addis Ababa: Ministry of Finance, 2006. Available: http://www.afdb.org/fileadmin/uploads/afdb/Documents/Policy–Documents/Plan_for_Accelerated_and_Sustained_(PASDEP)_final_July_2007_Volume_I_3.pdf. Accessed: 8 March 2016.

[3] Admasu K, Balcha T, Getahun H (2016). Model villages: a platform for community–based primary health care.. Lancet Glob Health..

[4] Ministry of Health of Ethiopia. Health extension program in Ethiopia. Addis Ababa: Ministry of Health, 2007.

[5] Godefay H, Byass P, Kinsman J, Mulugeta A (2015). Understanding maternal mortality from top–down and bottom–up perspectives : Case of Tigray.. J Glob Health.

[6] Balcha T, Getahun H, Admasu K (2015). Local innovations and country ownership for sustainable development.. Bull World Health Organ.

[7] Ghosh B. Non–doctor emergency surgeons are saving thousands of lives in rural Ethiopian hospitals. The Lancet Global Health Blog Available: http://globalhealth.thelancet.com/2016/01/13/non-doctor-emergency-surgeons-are-saving-thousands-lives-rural-ethiopian-hospitals. Accessed: 8 March 2016.

[8] Pharmaceuticals Fund and Supply Agency of Ethiopia, US Agency for International, Development. National survey of the integrated pharmaceutical logistics system. Addis Ababa: Pharmaceuticals Fund and Supply Agency of Ethiopia, 2014.

[9] Ethiopian Food, Medicines and Health Administration and Control Authority, World Health Organization. Assessment of substandard/counterfeit drugs in the Ethiopian pharmaceutical market. Addis Ababa: Ethiopian Food, Medicines and Health Administration and Control Authority, 2013.

[10] United Nations. Taking stock of the global partnership for development. New York: UN, 2015.

